# A transformative shift in urban ecology toward a more active and relevant future for the field and for cities

**DOI:** 10.1007/s13280-024-01992-y

**Published:** 2024-04-20

**Authors:** Niki Frantzeskaki, Daniel L. Childers, Steward Pickett, Fushcia-Ann Hoover, Pippin Anderson, Aliyu Barau, Joshua Ginsberg, Morgan Grove, Marleen Lodder, Ariel E. Lugo, Timon McPhearson, Tischa A. Muñoz-Erickson, Mien Quartier, Selina Schepers, Ayyoob Sharifi, Katrien van de Sijpe

**Affiliations:** 1 Department of Human Geography and Spatial Planning, Faculty of Geosciences, Utrecht University, Vening Meinesz Building A, Princetonlaan 8a, 3584 CB Utrecht, The Netherlands; 2 School of Sustainability, WCPH 442, Arizona State University, POB 877904, Tempe, AZ 85287-7904 USA; 3 Cary Institute of Ecosystem Studies, Box AB, Millbrook, NY 12545 USA; 4 Department of Geography and Earth Sciences, University of North Carolina Charlotte, 9201 University City Blvd, Charlotte, NC 28223 USA; 5 Department of Environmental and Geographical Science, University of Cape Town, Private Bag X3, Rondebosch, Cape Town, 7707 South Africa; 6 Department of Urban and Regional Planning, Bayero University Kano, PMB 3011, Kano, Nigeria; 7 Baltimore Urban Field Station, USDA Forest Service, 5523 Research Park Drive, Suite 350, Baltimore, MD 21228 USA; 8 Dutch Research Institute for Transitions, Erasmus University Rotterdam, Burg. Oudlaan 50, Mandeville Building, T16-42, 3062 PA Rotterdam, The Netherlands; 9 International Urban Field Station, International Institute of Tropical Forestry, USDA Forest Service, 1201 Calle Ceiba, Jardín Botánico Sur, Río Piedras, PR 00926-1115 USA; 10 Urban Systems Lab, The New School, 79 Fifth Avenue, 16 Fl., New York, NY 10003 USA; 11 Stockholm Resilience Centre, Stockholm University, Stockholm, Sweden; 12 Department of Environment and Sustainable Development, Stadsplein 1, 3600 Genk City, Belgium; 13 The IDEC Institute, Hiroshima University, 1-5-1 Kagamiyama, Higashi-Hiroshima, Hiroshima 739-8529 Japan

**Keywords:** Cities, Co-production, Transdisciplinary, Transformation, Urban

## Abstract

This paper builds on the expansion of urban ecology from a biologically based discipline—ecology *in* the city—to an increasingly interdisciplinary field—ecology *of* the city—to a transdisciplinary, knowledge to action endeavor—an ecology *for* and *with* the city. We build on this “prepositional journey” by proposing a transformative shift in urban ecology, and we present a framework for how the field may continue this shift. We conceptualize that urban ecology is in a state of flux, and that this shift is needed to transform urban ecology into a more engaged and action based field, and one that includes a diversity of actors willing to participate in the future of their cities. In this transformative shift, these actors will engage, collaborate, and participate in a continuous spiral of knowledge → action → knowledge spiral and back to knowledge loop, with the goal of co producing sustainable and resilient solutions to myriad urban challenges. Our framework for this transformative shift includes three pathways: (1) a repeating knowledge → action → knowledge spiral of ideas, information, and solutions produced by a diverse community of agents of urban change working together in an “urban sandbox”; (2) incorporation of a social–ecological–technological systems framework in this spiral and expanding the spiral temporally to include the “deep future,” where future scenarios are based on a visioning of seemingly unimaginable or plausible future states of cities that are sustainable and resilient; and (3) the expansion of the spiral in space, to include rural areas and places that are not yet cities. The three interrelated pathways that define the transformative shift demonstrate the power of an urban ecology that has moved beyond urban systems science and into a realm where collaborations among diverse knowledges and voices are working together to understand cities and what is urban while producing sustainable solutions to contemporary challenges and envisioning futures of socially, ecologically, and technologically resilient cities. We present case study examples of each of the three pathways that make up this transformative shift in urban ecology and discuss both limitations and opportunities for future research and action with this transdisciplinary broadening of the field.

## Introduction

The field of urban ecology has long been focused on addressing classic ecological questions in urban landscapes—what has been called “ecology in cities”—and in the last decades, it has expanded to include more interdisciplinary and holistic social-ecological approaches—an “ecology of cities” (Pickett et al 1997; Grimm et al. 2000; Lin and Grimm 2015). More recently, an additional expansion of urban ecology has acknowledged that the field now includes a more “hands-on” transdisciplinary approach that is focused on real-world solutions as well as basic research—an “ecology for cities” (Childers et al. 2015) or “ecology with cities” (Pickett et al. 2022). We conceptually position urban ecology as a broad field that is informed by inter- and transdisciplinary research and practice with diffused boundaries.

**Fig. 1:**
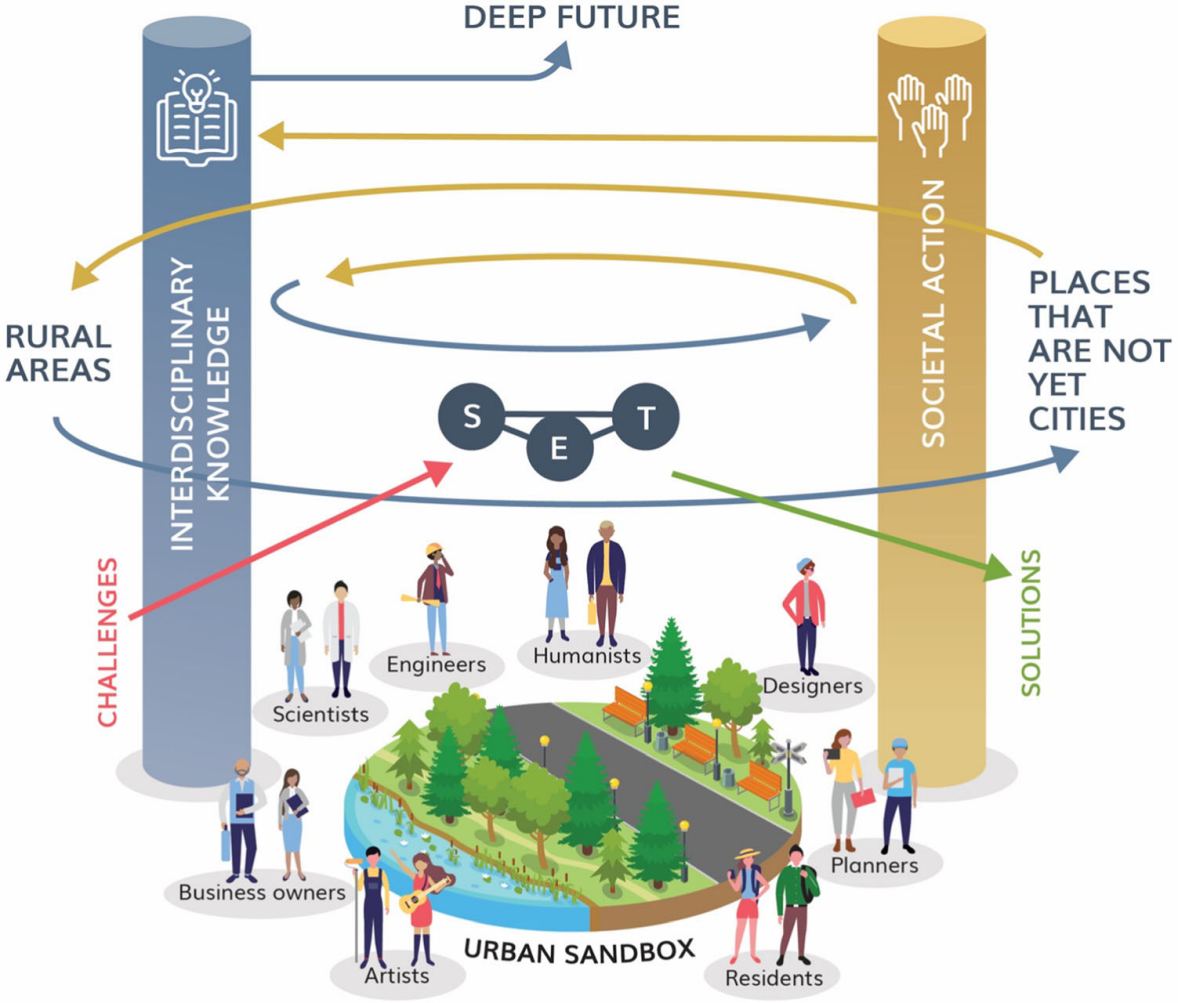
The transformative shift framework. In this transformation of urban ecology, a diverse collection of actors work together in what we call an “urban sandbox” (bottom) to address societal challenges in a constant, iterative knowledge → action → knowledge loop or spiral (center). This interdisciplinary and equity-driven process of generating knowledge to solve problems includes social, ecological, and technological aspects of the urban form, but it also involves consideration of broader, non-urban issues and challenges (rural areas and places that are not yet cities). A key goal of this transformative shift to produce positive societal action is focus on not just tomorrow, but also on decades into the future, or what we call the “deep future” (top)

We argue that the myriad challenges facing cities and societies today and into the future require a more involved, even activist, urban ecology that is centered on constant feedback of interdisciplinary knowledge to action, and back to knowledge. In this paper we present a framework including three potential pathways by which the field of urban ecology may experience a transformative shift toward a future of solution-oriented action and enhanced relevance. The need for this shift is supported by evidence of a gap between available empirical urban ecological knowledge and the urban design, planning, policy, and future visioning that it should be informing (Nesshover et al 2017; Gagné et al. 2019; Wang et al. 2021; Raska et al 2022). This transformative shift will position the field of urban ecology as an agent of change for society by ensuring that knowledge production, translation, and application are central, more meaningful (socially and ecologically), and more impactful for both science and society. This centrality of knowledge → action → knowledge feedback is the backbone of our transformative shift framework (Fig. 1), which we describe below, and which presents a paradigm shift in the field to one that actively engages and informs ongoing policies and urban dynamics. As such, we conceptualize that urban ecology is in a state of flux with a continuous co-evolution of concepts, knowledge → action → knowledge feedback loops, and engagement with practitioners and communities.

The three pathways that we propose to facilitate this transformative shift in urban ecology, and that we depict in our framework (Fig. 1), are: (1) a cyclical, reciprocal knowledge → action → knowledge process of ideas, information, and solutions taking place among a diverse community of agents of urban change—we call this the knowledge → action → knowledge spiral—that extends beyond the feedbacks as generally defined in co-production; (2) incorporation of a social–ecological–technological systems (SETS) framework and expanding the spiral upwards, or temporally, to include [what we call] the “deep future,” where building future scenarios allows the visioning of previously unimaginable future states of cities that are sustainable and resilient; and (3) the expansion of this spiral sideways, or in space, to include rural areas and systems (sensu the Continuum of Urbanity; Boone et al 2014; Zhou et al 2021) as well as places that are urban in character, but that are not yet cities. We unpack these three transformative shift pathways in “Unpacking the transformation shift” section.

Our framework for these transformative shift pathways begins with a diverse community of actors who work toward and/or are interested in sustainable and resilient transformation for cities. These agents of change include [among others] scientists, engineers, designers, planners, residents, artists, business owners, and activists. We envision and hence conceptualize a transformative shift in which urban ecology is pursued by more than just urban ecologists. In Fig. 1, we situate them at the bottom of the knowledge → action → knowledge spiral in an “urban sandbox”. This is a metaphor for the sandboxes often found in playgrounds, where children can come together to play and cooperate; places where collaboration conquers conflict. We also draw inspiration from the regulatory sandboxes, as spaces where different actors come together to set courses for innovation in a ‘constructed absence of regulation,’ with the focus on collaborative or co-created innovations (Beckstedde et al. 2023). In our conceptualization of the urban sandbox, we position the voluntary engagement of all interested actors as critical to contributing to sustainable and resilient transformations in cities. On either side of the sandbox are the pillars of interdisciplinary knowledge and societal action, with the spiral being constant iterations between them. With this continuous spiral of feedback loops, interdisciplinary knowledge is dynamic, builds continuously from social actors’ interactions and weaved knowledges, and continuously interacts, shapes, and is shaped by societal action and vice versa. Embedded within this spiral are the ecological, social, and technological systems, also known as SETS, that make up the city's infrastructures. Challenges and problems are constantly spinning into the knowledge → action → knowledge spiral, which spins out sustainable, resilient solutions. Where the spiral expands beyond this urban SETS realm, it is incorporating rural areas and systems and places with urban characteristics but that are not yet cities. And the spiral spins out of the top into “deep futures” (Fig. 1). In this transformation of urban ecology, a diverse collection of actors work together in what we call an “urban sandbox” (bottom of Fig. 1) to address societal challenges in a constant, iterative knowledge → action → knowledge loop or spiral (center of fo rFig. 1). This interdisciplinary and equity-driven process of generating knowledge to solve problems includes social, ecological, and technological aspects of the urban form, but it also involves consideration of broader, non-urban issues and challenges (rural areas and places that are not yet cities). A key goal of this transformative shift to produce positive societal action is focus on not just tomorrow, but also on decades into the future, or what we call the “deep future” (top of Fig. 1).

To develop this transformative shift, we draw from the literature on ecosystem services, urban ecological infrastructure, and nature-based solutions to illustrate how the transformative shift propagates and the conditions required to facilitate it. The ecosystem services concept—alongside alternative conceptualizations such as the contributions of ecological systems to people (Hill et al. 2021)—is a conduit to renaturing the design and evolution of urban form. A key challenge is that this concept is lesser known and understood outside academic circles (Brink et al. 2018; Elliot et al. 2020; De Luca et al. 2021). This knowledge gap further corroborates the need for a more inclusive and interconnected epistemological pathway between science and practice in urban ecology. For example, urban ecological infrastructure (Childers et al. 2019) is a more inclusive term than green infrastructure because it includes all urban components that support ecological structure and function in cities, not just those that are explicitly designed, constructed, or managed for human uses (sensu Barau et al. 2013, 2020). Rethinking people’s connections to ecological systems and efforts to curb biodiversity loss will require a more action-oriented and solution-driven perspective in urban ecology. We describe the three pathways of this ongoing (and needed) transformative shift in “Unpacking the transformation shift” section, and we address both the challenges of this transformation shift and the opportunities it presents further in “Challenges and opportunities of the transformative shift in urban ecology” section.

## Unpacking the transformation shift

In this section, we explore three pathways that capture how the transformative shift is being manifest or simply how the field is already moving toward an inclusive and open research paradigm. We also propose a direction for progressing urban ecology farther by bringing other disciplines on board.

### Pathway #1: Embracing the knowledge → action → knowledge spiral

Moving from a linear model of knowledge to policy and civic action toward a more interactive mode of how urban ecological knowledge connects to, builds from, and is enriched action is the first proposed pathway of the transformational shift. Specifically, this is a fundamental shift in urban ecology from knowledge-generating research to a knowledge-to-action-to-knowledge enterprise. As we conceptualized in the urban sandbox model (Fig. 1), there are continuous interactions and feedback among the diverse knowledges that urban change agents bring to social action, co-producing a spiral of connected interactions. Expertise and experience gained through participatory action research or community-based participatory research proffers valuable lessons on how to grow, expand, and open the knowledge inquiry process in urban ecology. This pathway considers that in every scientific inquiry into real-life social systems, such as urban systems, knowledge production requires social validity that comes from consultation and collaboration with willing social actors. While the co-production of knowledge in urban ecology is not a new concept (Cadenasso and Pickett 2018), it is receiving increased attention as a mode of active engagement of diverse actors in framing social conditions for transformative and sustainable urban solutions (Frantzeskaki and Kabisch 2016; Kabisch 2019; Visconti 2023; Wickenberg 2023). We argue that the continuous interactions between knowledge inputs and social action must be both conceptualized and grounded in empirical experiments and evidence.

Pickett et al. (2021) posited that ecology with the city is inherently transdisciplinary and requires interaction and co-production among scientists, decision-makers, regulators, and residents. Building from this understanding, they noted that the “coproduction of just, actionable knowledge […] can be used in equitable ways for planning and managing the city”. Andersson et al. (2022) argued for the need for multi-sector and multi-actor engagement in planning for nature in the city “to better account for non-physical constraints, user perspectives and diversity among users.” Schaefer (2022) noted the value of co-production for increasing understanding, respect, and trust among scientists, planners, and other decision-makers, and for urban ecologists to reflect on their roles in the process. Marshall et al. (2020) also showed how a knowledge-to-action feedback loop can be organized to facilitate the weaving of various stakeholder interests, perspectives, and knowledges toward a ‘patch atlas’ tool and atlas ecological urbanism model.

Similarly, recent research on nature-based solutions also points to the importance of recognizing and facilitating the feedback loops connecting communities, planners, and scientists (Collier et al 2023). Wellmann et al. (2023) pointed to the need for inclusive language and transparency in interactions, so these knowledge feedback loops are acknowledged, and to ensure that equal footing is given to expert and non-expert (often tacit, local, and/or Indigenous) knowledge. In this co-production pathway, communities and stakeholders include citizens, community organizations, and social enterprises alike, with their degree of engagement varying depending on the planning issue or challenge and on the tacit knowledge and experience needed to co-design and co-produce a solution. Zhou et al. (2021) noted that “urban ecosystem research is often justified by practical concerns” in their argument for a transdisciplinary urban ecology as a basis for a science of cities. For this pathway to progress, new institutional approaches need to be created by shifting ways of thinking about the knowledge production enterprise to one of multi-actor engagement and for deconstructing social–ecological injustices in how knowledge is employed for decision-making (Muñoz-Erickson et al. 2017).

#### Space for multi-actor engagement, activation, and co-production

This transformational pathway needs to create and advocate for the inclusion of urban residents as stakeholders in decision-making processes and processes of city-making. This is not a common practice in urban planning, even though there is considerable evidence of the value of ecological knowledge and perceptions of residents in shaping urban infrastructures. Modes of knowledge production such as co-design, and co-production already demonstrate how 360 degrees of knowledge generation is possible, albeit challenging and not immune to concerns about selective inclusivity or even intentional exclusivity. Grabowski et al. (2023) provide an analysis of 120 relevant green infrastructure plans in 20 U.S. city documents via text analysis that shows that equitable involvement of residents and communities in the planning apparatus is, in fact, rare. Current research notes the importance of multi-actor engagement, that regardless the effort put in especially in the European context (Collier et al. 2023), it is not (yet) a common practice. Wellmann et al. (2023) pointed to the importance of incorporating input from local communities when planning and designing nature-based solutions, moving to local knowledge integration for locally embedded nature-based solutions. Such local knowledge integration for locally embedded nature-based solutions aligns well with theory and cases studies on collaborative planning for nature-based solutions in cities (Vano et al. 2021). As Connop et al. (2016) pointed out, the understanding and consideration of local context needs to lead the design and development of local biodiversity interventions. Adding an intergenerational perspective to this, Grey et al. (2023) pointed to the importance of considering aging communities and individuals in the co-production of knowledge and place for ecological urbanism.

Shared spaces for knowledge co-production among diverse and willing actors, such as the urban sandbox show in Fig. 1, function to reconfigure power relations embedded in traditional systems of knowledge production (Patel 2022). An important aspect of these shared, neutral spaces is the need to decenter academia in the co-production of knowledge (Alonso-Yanez et al. 2019). Thinking from the urban sandbox (Fig. 1), that may mean that in some urban contexts, citizens may be the ones starting the feedback loop of knowledge to action to knowledge and start with shaping transformative solutions to urban challenges with scientists, joining the urban sandbox in later stages of the co-production process. This means that the co-production of knowledge can and should take place without the prominence of academic knowledge, while not neglecting its value (Rademacher et al 2018).

The democratization of the knowledge enterprise of urban ecology has recognized value for urban planning and for society. Moving to open modes of knowledge co-production allows for power redistribution and new relationships to be created (McHale et al 2018; Woroniecki et al. 2020). Going beyond advocating the importance of co-production to advancing approaches and tools to achieve co-production has been an advancement of the field in recent years. Zhou et al. (2021) showed how a plurality of theories, when integrated into the meta-city concept, can facilitate a transdisciplinary pathway for urban ecology. Co-production approaches have been trialed, designed, and evaluated in different transdisciplinary settings (as designs) such as urban living labs (Frantzeskaki 2019; Barau et al. 2020; Mahmoud et al. 2021) and participatory scenario development (Cook et al. 2021; De Luca et al. 2021; Cook et al. 2022).

An example of democratizing knowledge is citizen science, which involves citizens collecting, documenting, or broadly being active in the data collection phase of research that also improves or enriches ecological literacy (Bonney et al. 2009). Citizen science has shown potential in actively and openly engaging with citizens in the knowledge production process (Conte et al 2023) that is an iterative, communication process (Bruckermann et al 2022). Greving et al. (2023) argue that citizen science can achieve a sense of pride and responsibility for contributing to urban ecological conservation, which further relates to awareness of urban wildlife. As with any open engagement approach, citizen science is not immune to challenges and limitations. Bonney (2021) specifically points out that citizen science field is increasingly more criticized about equity, diversity, and inclusion in the way volunteers are recruited and explains the challenges that come with such an open approach to inquiry whereas recognizing the need for citizen science to “address historic inequities that have limited whose knowledge is valued by and represented in both academic research and regulatory monitoring.”

An illustrative case is presented in Box 1, which overviews the different formats of co-production used by scientists, planners, and citizens in the City of Genk, Belgium, in the context of an urban regeneration project for the Stiemer stream and valley.

## Box 1: Re-connecting people–nature through co-production in the City of Genk, Belgium

The city of Genk, Belgium, has been undertaking urban regeneration of the Stiemer Stream over the past 10 years. The banks of the stream were primarily paved and suffered from littering and sewage overflows because the city had turned its back on the Stiemer many years ago, effectively stigmatizing any effort to rejuvenate the area (Tractebel et al 2019). By envisioning the Stiemer and its surrounding valley as a connector of neighborhoods and social groups, the city officers/planners of Genk were able to co-design a new planning process that engaged scientists, citizens, and entrepreneurs in different forums and formats. Four co-production formats generated the most impactful outcomes in terms of ideas, new senses of place and belonging of the communities, and nature-based enterprises, meaning local small-medium enterprises that stewarded part of the valley for its protection and for deriving socio-economic benefits from its regeneration such as beekeeping, eco-tourism activities, and nature management (Hill 2022). These four co-production formats are:

(a) *Co-design workshops with scientists, urban planners, and citizens*: For the planning and implementation of the Stiemer Valley large-scale regeneration project, the city of Genk engaged scientists and other experts on nature-based solutions as systemic interventions to help regenerate the valley. From 2014 to 2022 14 of these co-design workshops took place, focusing on different planning needs, such as the identification of nature-based solutions for sustainable urban water drainage systems and urban trees, engagement with citizens to identify desires and needs for infrastructure for recreation and cultural events, and monitoring and evaluation co-production workshops to assess progress and outcomes. These workshops produced two long-term co-design trajectories (‘Junior Team’ and ‘Waterrijk Waterschei’), of which the latter is still ongoing. An example of such a co-design trajectory is the Junior Team process—set up by the city's Youth Department and Environment department together with a local university college—that involved twelve 10–12 years-old children to envision the future of the Stiemer valley, raising their awareness and knowledge while co-developing ideas on how to make the valley attractive. Three resulting ideas were realized and implemented, including a treehouse (Hölscher et al. 2020; Schepers et al. 2019)

(b) *Stiemer Deals*: Stiemer Deals are tailor-made agreements among the City of Genk and other stakeholders (e.g., individual citizens, organizations, private companies) in which the objectives of all parties are pursued, resulting in a win–win situation for both. The Stiemer Deals concept embodies a social innovation strategy that enables multiple and diverse actors to feel ownership over the Stiemer Valley planning process. The concept aims to accelerate the socioeconomic transformation of the valley. An example of a Stiemer Deal outcome is a local organization for psychologically vulnerable people that partnered with a Flemish nature organization to adopt and manage a grassland habitat in the Stiemer Valley (Hölscher et al. 2022)

(c) *Friends of the Stiemer Group:* Created in November 2018, this is a group of engaged citizens that act as ambassadors for the Stiemer process and mediate between the city government and citizens. This initiative epitomizes structural, ongoing communication and participation in the Stiemer Valley process. The 'Friends of the Stiemer' cooperate with the city administration and external experts in a citizens' panel that follows the progress of the long-term urban regeneration process. The group generates goals and ideas for implementation, mobilizes actors to participate, informs about citizen agendas, and co-produces communicative initiatives and events. They meet two to four times a year and are regularly updated between meetings by the city of Genk on the progress of the Stiemer Valley regeneration (Hölscher et al. 2022)

(d) *Reflexive monitoring*: The co-production process was supported by a learning-by-doing and doing-by-learning process based on Reflexive Monitoring (Beers and van Mierlo 2017; Dentoni et al.^2016^; Frantzeskaki et al 2016). For 3 years (2018–2020) this process was supported by the researchers of the Connecting Nature project, who applied the scientific method with the city team to organize themselves (see guidebook: https://oppla.eu/product/23324, pp. 70–73). This process helped the team record and track what they were learning over time and analyze the learning process. This allowed them to connect short-term actions to the long-term transformative goals of the program. The learning outcomes were analyzed for their contributions to the establishment of new rules, relations, practices, and discourses based on Beers and van Mierlo (2017). Having explicit learning outcomes helped the city team communicate project results with project outsiders. The city team embedded this learning process into the governance structure for the Stiemer Valley program, linking strategic long-term goals to operational short-term actions, and are using it for other complex projects

**Figure:**
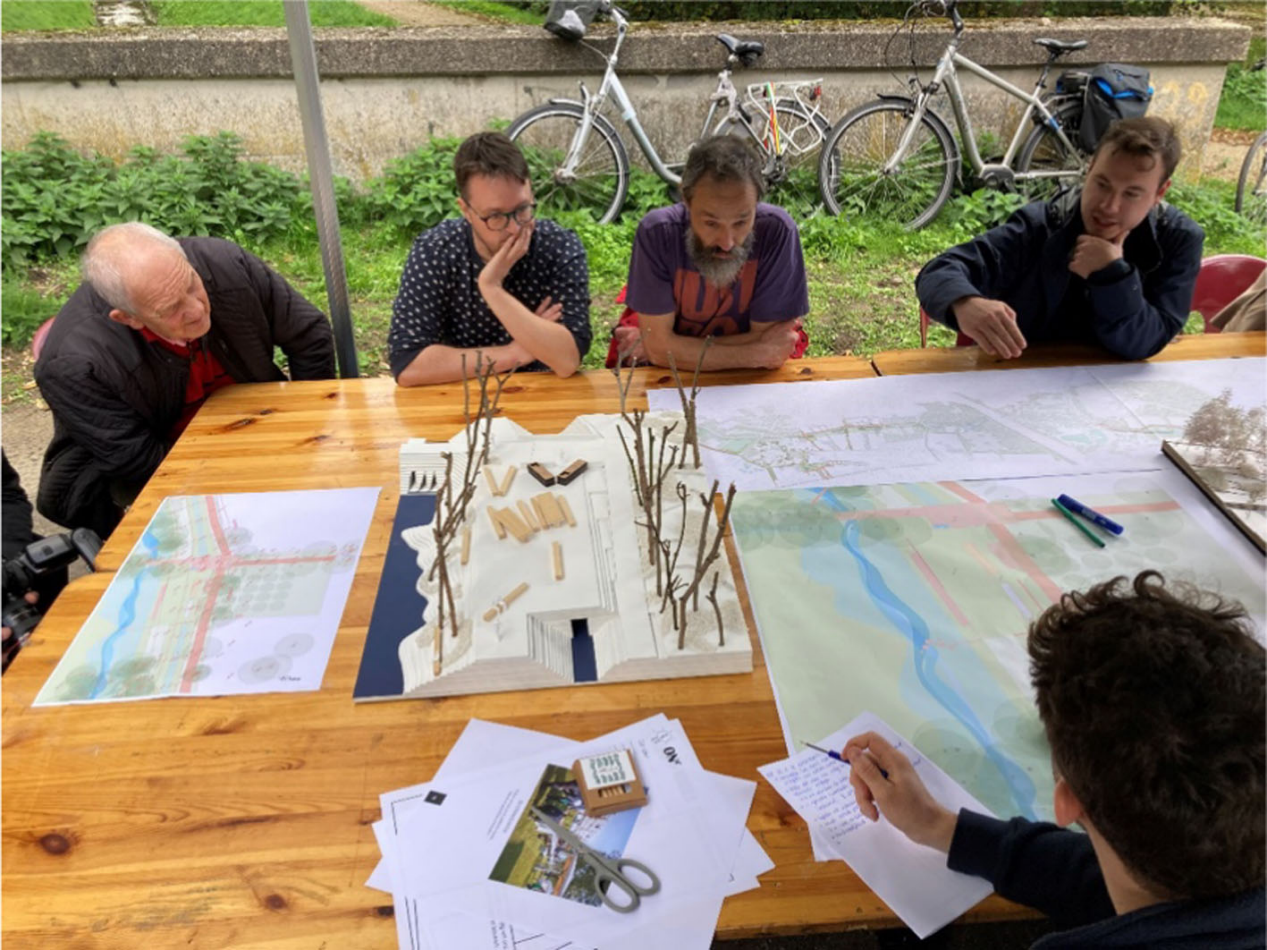
Photograph 1: Example of a co-design workshop in October 2022: citizens, architects and employees of the city’s Environmental department generating ideas on a specific site in the Stiemer Valley, Genk, Belgium. Photo credit: City of Genk

The Stiemer Valley regeneration process, as co-designed and co-produced by urban ecologists, urban planners, designers, and citizens, demonstrated that different types of institutional settings are required and are possible for including and empowering multiple actors in the city. Co-production processes tend to be time-intense and demanding, requiring institutional creativity and flexibility from local administration and skills to develop them, so documenting their outcomes, value, and impact is critical

### Courage to recognize, unpack and remedy social–ecological injustices

Opening the scientific inquiry of urban ecology to planners, civilians, and other knowledge holders will create opportunities to address and remedy the legacies of past social and environmental injustice, making cities and their design, management, and futures equitable and just. Grabowski et al. (2023 p. 3) noted that “planning is also a contested arena in which the rules governing urban systems can be rewritten in collaboration with marginalized communities to achieve equitable transformations.” That further points to the opportunity for restoring justice through collaboration and co-production in planning between planners, scientists, and communities. The first efforts in this work can and should be acknowledgment and acceptance of how our urbanized landscapes are racialized and have been structured to create and maintain unjust social-ecological relationships (Hoover and Scarlett 2023). Unpacking and addressing these legacy and current unjust relationships requires understanding personal experiences and connections to space and place (meaning understanding place-making) and prioritizing community-led and community-engaged civilian science, activism, and planning (Raymond et al 2023). In this vein, the Bronx River Alliance presents a real-world example of how this broadening of the scope of urban ecology may be realized. This case (Box 2) depicts an alliance that unroots injustices while broadening the focus to an urban ecology with and for people and nature.

## Box 2: The Bronx river alliance

The Bronx river alliance (BRA) is one organization that integrates research and activism to address ecological concerns and environmental injustices. Founded in 2001, the BRA, formally the Bronx River Working Group, grew out of community activism and engagement efforts with Partnership for Parks. Representing one of the most diverse boroughs in New York City (NYC), the Working Group consisted of 30 different groups from across the borough, all focused on the Bronx River as a resource. It brings key communities together to develop a vision for the river (e.g., open space, ecological goals, education, connectivity, outreach). Meeting semi-annually, the Working Group is comprised teams focused on different aspects of the Bronx River and adjacent community needs like housing, arts, or public services. It is important to note that the Bronx River flows from Westchester County (one of the wealthiest counties in New York State and the U.S., into the Bronx (the poorest region in the State of New York). Despite being a community-initiated organization, BRA was a majority-white-led organization by 2005. In response to this and continued feedback from the BRA teams and community members for staff who reflected the community, Executive Director Maggie Greenfield spent the next 10–15 years hiring through local recruitment to fill internships, entry-level organizing positions, and volunteer staffing, promoting internally. A leadership and coordinator team that was once all white now has a 40% white leadership team and staff that is 90% people of color. As Ms. Greenfield noted, “The mission is also felt more deeply by members in the community,” which strengthens the organization and ensures longevity

An important facet of how BRA practices urban social ecology is through its programming and community science, specifically Project Waste, Project Water Drop, and Ecoteams. These programs engage residents by asking them to collect and contribute data on the river, including concerns or issues they see in their neighborhoods, experiences, and priorities. Through these Ecoteams, reports, and presentations on Rivers are drafted with input from teams, the semi-annual assembly, and public meetings on a watershed plan. An engineering firm then brings together this information and input to help inform priorities. One example of this process was the 2010 Inter-Municipal Joint Plan, published in collaboration with the NYC Parks and Westchester County; the plan incorporated biology, engineering, community perspectives, lived experiences along the river, and various stakeholder engagement. Since its formal founding, BRA has worked to maintain community leadership and to create visioning and management plans that address the river’s water quality problems, experiences with the river, and other community needs

### Pathway #2: Embracing the SETS framework and expanding the spiral into the “deep future”

Much of the infrastructure that is designed, constructed and managed to make cities more resilient is highly engineered and technocentric. In this pathway, urban ecology expands its social-ecological focus from “nature in the city” to explicitly embrace the built environment with a robust social–ecological–technological systems paradigm that reconciles and considers historical context and contemporary conditions, such as politics, to evolve the field (Pickett and Cadenasso 2008). Markolf et al. (2018) argued that this single-focused approach to stability in urban systems makes cities more vulnerable to infrastructural failures by creating a false sense of security (Chester et al. 2023), and that urban infrastructure should be viewed as complex and interconnected social–ecological–technological systems (SETS; Grimm et al. 2015; McPhearson et al. 2016, 2021, 2022). The SETS literature is growing rapidly, and includes scholarship on the relationship between urban SETS and disturbance (Grimm et al. 2017; Lugo 2019, 2020), the use of SETS to reduce urban flood risk (Chang et al. 2021), enhancing positive SETS feedbacks to address heat- and drought-induced stresses on urban ecosystems (Wellmann et al. 2023), the governance and environmental justice implications of urban SETS (Pineda-Pinto et al. 2021; Krueger et al. 2022), and the multifunctional ecosystem services provided by SETS (McPhearson et al. 2022). Branny et al. (2022) drew on this literature to present a systems approach for "smarter, greener" cities that utilizes SETS-based integrated solutions rather than more traditional, single-dimensional, technology-heavy solutions. Chester et al. (2020) made a strong case for the vulnerability of exclusively engineered infrastructure in the face of a future of uncertainty. Recent research on nature-based solutions as systemic solutions for building urban climate resilience (Frantzeskaki et al. 2019) has argued for adoption of a SETS approach to systematically understand how implementation of greening or renaturing programs, and urban ecological infrastructure, may be realized (Wellmann et al. 2023). In the same vein, SETS has informed diagnostic studies of urban injustices, and has been used to integrate concepts from ecological justice, urban ecology, and post-humanism (Pineda-Pinto et al. 2021). The most robust solutions to urban resilience challenges are likely to come from multidimensional SETS approaches (McPhearson et al. 2016). For this to be realized, the knowledge → action → knowledge spiral will provide inclusive learning spaces to co-produce SETS knowledge and inter- and transdisciplinary research designs and practices. As Feagan et al. (2023) stated: “SETS knowledge co-production requires a context-specific pedagogical design for interrupting dominant power relations to allow new knowledge-sharing practices to emerge.”

Using the SETS framework and approach to develop scenarios by weaving different knowledges and perspectives is one way to expand the knowledge → action → knowledge spiral in time and into “deep futures”. Cities are spatial entities, and urban ecology has long been a science of spatial variation, with considerably less emphasis on temporal variability. The concept of dynamic heterogeneity (Pickett et al. 2017) effectively bridges space and time, and Ossola et al. (2021) argued for a stronger emphasis on temporal dynamics in urban systems science. Their concept of an “urban chronos” (*chronos* in Greek means time, introducing the temporal dimension) is focused on changes in urban ecosystems over time, which is the first bridge to considering the “deep future” of cities and urban systems. We argue that using scenarios and future visioning, and their fully inclusive and equitable development, is an excellent forum for thinking about urban systems with a long temporal vision. In their review of the recent history of scenario planning, which was not explicitly urban-focused, Varum and Mello (2010) noted the importance of including both researchers and practitioners in the scenario planning process. Dixon (2022) presented a framework for urban science research that highlights the importance of the built environment—the “T” in SETS—when addressing [what he referred to as] urban systems science and sustainable urban futures. In other words, the development of scenarios and future visions must, from the start, include planners, designers, and engineers. Other authors have argued for the importance of normative approaches to futures visioning exercises, particularly when the goal is more sustainable and resilient urban systems (Pelling et al. 2023). There are several recent examples in the literature of this type of normative, values-based, co-produced future scenarios for cities, and an example that is proximal to several of the authors of this paper is Iwaniec et al. (2020). Finally, and perhaps most resonant with the goals of this paper, is the recent work by Mansur et al. (2022) on nature futures for cities. Their approach calls for a melding of fully participatory visioning exercises with quantitative models that are focused on urban social–ecological feedback, assessing the indirect effects of cities on biodiversity, and the use of multi-scalar indicators and future scenarios. Components of all such approaches should be used by urban ecologists as we strive to extend the knowledge → action → knowledge spiral into deep urban futures in the most inclusive, equitable, and just ways possible, and Box 3 is an example of this.

## Box 3: New York city’s climate adaptation scenarios

New York City has created a wide range of hazard mitigation, emergency response, and climate adaptation and mitigation plans over the last decade and more. However, there are no plans that look beyond 2050, or into the “deep future,” and very few plans that examine the multi-hazard context that is already affecting people, infrastructure, and ecosystems in the city in the face of climate and weather-related extreme events. As part of a National Science Foundation-funded *Converging SETS for Urban Resilience* project, a team at The New School, Barnard College, Georgie State University, Arizona State University, and the USDA Forest Service developed and facilitated a process to allow diverse stakeholders across multiple levels of city government to collaborate in a series of workshops designed to enable the development of visions, scenarios, goals, targets, and strategies for delivering a resilient, equitable, and sustainable New York City by 2100. This was a truly deep future approach

Approximately 35 government practitioners from 24 of all relevant city, state, and federal agencies gathered virtually over the course of 5 weeks. Together, participants co-developed six distinct climate adaptation scenarios. The goal of each future scenario was to radically transform the social, environmental, and physical infrastructure of the city—including governance, UEI, and water-energy-transit systems—as well as the ability to respond effectively to extreme events. Participants worked in small groups to envision six scenarios for resilient futures of New York City in 2100. The envisioned future scenarios addressed multiple co-occurring hazards, coastal flooding, extreme heat, winter extremes, extreme precipitation, and drought and shifting water demand (Cook et al. 2022)

**Fig. 2:**
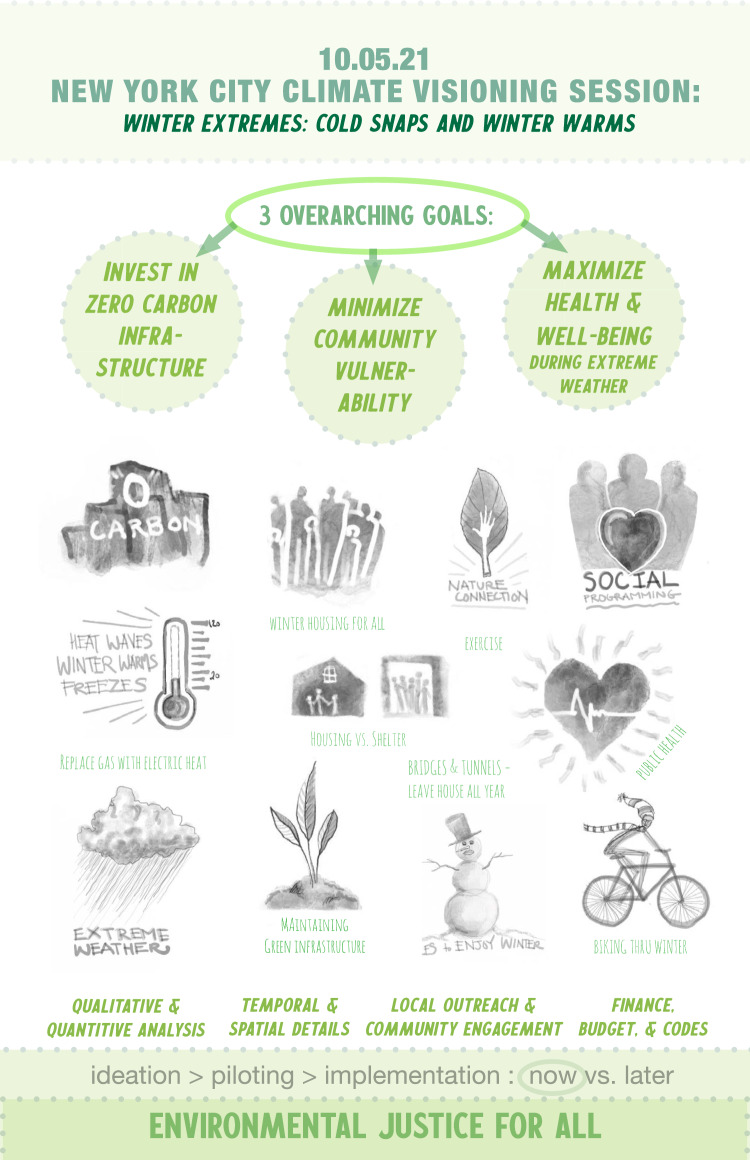
Winter extremes scenario visualization by Artist Ann Armstrong for the NYC Adaptation Scenarios (Cook et al. 2022)

Scenario themes were developed in response to practitioner concerns and existing sustainability and environmental management plans. Activities included innovative ideation, timelines, visual illustrations, and day-in-the-life narratives, and participants defined long-term goals and strategies for each scenario to develop radical deep future visions for New York City in 2100. Existing NYC climate governance and strategies were seeded as a starting point to inform scenario development and build on visionary work already happening in the city. This scenario and future visioning development exercise used the SETS conceptual framework to guide visioning processes, including ensuring that all participants considered social, ecological, and technological aspects of both challenges and solutions. Additionally, taking the systems approach forward meant examining SETS couplings (McPhearson et al. 2021; Branny et al. 2022). This meant examining the social–ecological (S–E), social–technological (S–T), and ecological–technological (E–T) dimensions, dynamics, and feedbacks of climate impacts and deep future solutions (Fig. 2)

### Pathway #3: Expanding the spiral to include places that are not yet urban

It is important that urban ecologists think beyond merely places that are urban today and recognize what can be learned about and from places that are on their way to becoming cities as well as place that are not yet urban. These places may be towns or villages that are likely to be cities in the future; but they may also be places with urban characteristics but that are clearly not what would be classically defined as cities (sensu McHale et al. 2015). Smaller or newer cities and towns/villages that are not yet cities are far less burdened by systemic inertias—infrastructural, institutional, and social—than are existing cities (Childers et al 2014). We argue that these new cities, and places that are urban but are not yet cities, are prime venues to demonstrate that transformation in urban forms is desirable and possible (Childers et al. 2015) and that it works to enhance resilience (Andersson et al. 2022). Thinking from the urban sandbox perspective, in these places all actors need to and can contribute their knowledge and understanding of social and place dynamics while co-shaping the ideas and solutions attuned to the future of their localities. The new knowledge that will emerge from these transformative solutions should also help guide and inform new growth and rehabilitation in existing cities.

A context for this transformative shift in urban ecology, to considering places not yet cities, is the urban–rural comparison/contrast history of the discipline. Urban–rural gradient analyses, comparisons, and inter-dependencies have a long history in urban ecology. Researchers such as Gutierrez-Vélez et al. (2022) have argued that these conceptual and empirical approaches have always had a strongly urban-centric focus and that a re-centering of urban–rural thinking should be (re)conceptualized. For such a recentering, connectivity and interconnectivity are key to moving away from a strictly urban-centric approach to thinking beyond existing cities. There is clear justification that we live in an urban century. The majority of people currently live in cities, and in the future many more people will live in urban areas. Urban systems are simultaneously viewed as either the innovation saviors of sustainability and resilience, or the source of the largest challenges we are facing (Artmann et al. 2019; Seto et al. 2012). An example of expanded urban thinking is the conceptual framework Jamshed et al. (2020) presented on how rural–urban dynamics are linked to flooding vulnerability in rural communities. Tools such as this framework will help urban systems researchers understand multifaceted rural vulnerability and its dependence on the urban systems to which these rural areas are linked and networked. However, we posit that urban ecologists need to think about rural systems from the standpoint of places that are not yet cities. Boone et al. (2014) presented a theoretical and conceptual construct called the “continuum of urbanity” that helps bridge this rural–urban divide. In Box 4 we present an example of how this continuum of urbanity concept can be expanded to think beyond places that are merely rural today, and to include places that may [or may not] be currently urban but that are clearly not yet cities.

## Box 4: Places that are not yet urban

One of the hallmarks of the transformative shift is to focus beyond areas that are currently identified as cities in particular or as urban in general. This expansion of the focus on the nature of urban places and life beyond the official limits, or the familiar human density, infrastructural intensity, and concentration of resources and economical consumption, was highlighted by Henri Lefebvre (originally 1968, trans-2003). He spoke of how the urban, including its influence on industrial agriculture, eroded, and subsumed rural livelihoods and village life. Many scholars have documented the increasingly regional and global reach of urban characteristics and processes. City limits and municipal boundaries are no longer an adequate focus of attention on urban phenomena. For example, rural areas of the Amazon now experience connections of demand and influence from urban centers (Brondizio et al. 2016). Similarly, large discount stores and online shopping insert urban aesthetics and amenities into small towns and homes distant even from villages and hamlets (Güneralp et al. 2013). And tourist rentals and vacation homes insert urban lifestyles, leisure habits, new service jobs, and capital into formerly agricultural or pastoral landscapes, displacing employment and populations tied to the land (Hof and Blázquez-Salom 2013)

The Yanqi Valley example we present here is based on collaborative research among co-authors Childers and Pickett and Weiqi Zhou, from the Chinese Academy of Sciences, Beijing. We focus on the transformation of this rural valley on the fringe of Beijing, China, from a formerly agricultural and orchard landscape embedded in a forest matrix into a tourist destination. With better connectivity to the increasingly well-off population of Beijing and connection via the internet to vacationers from other countries, a tourist-based economy is rapidly supplanting the past dominance of farming. The figure here shows the 94 km^2^ watershed of the Yanqi Valley, with hypotheses of some variables that index the increasing urban influences on this once and seemingly rural watershed. Notably, these hypotheses are framed using the Continuum of Urbanity, mentioned earlier
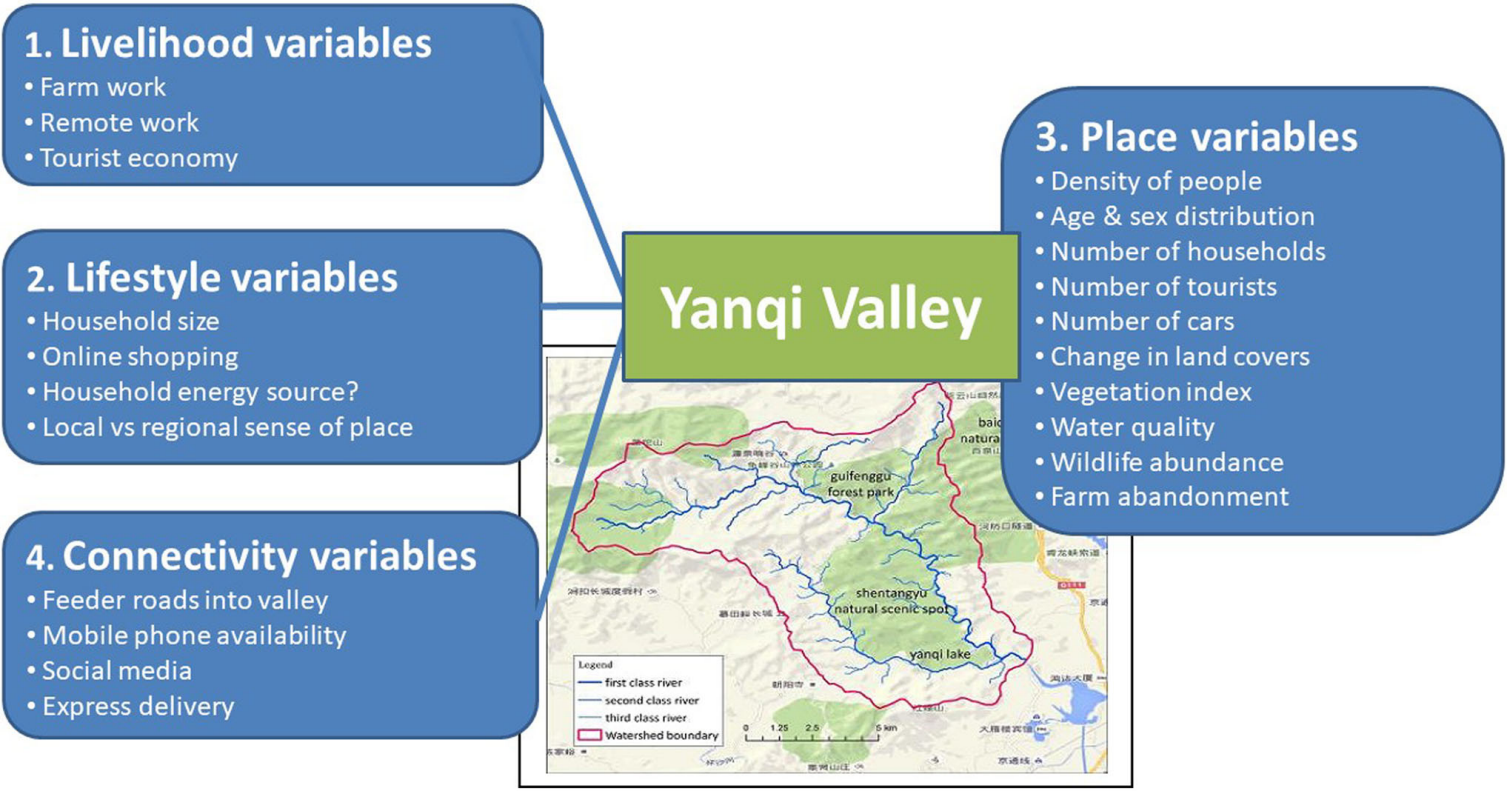


These hypotheses emphasize that with the increasing pressure and influence from leisure-seeking urbanites, the following changes have been taking place: (1) *livelihoods* of valley residents shift from farming and orcharding to service in a tourism industry; (2) *lifestyles* of local residents become more oriented around consumption rather than agricultural production; and (3) the local ecosystem or *place* becomes biotically and biogeochemically altered and likely degraded. All these changes flow from the increasing ease of (4) *connectivity*, including information, resources, and actual travel, that the Yanqi Valley experiences with the influx of regional and international consumers of “rural” experiences. This is an excellent example of urbanity beyond the boundaries of a city. Indeed, in response to these pressures, the Yanqi Valley is likely transforming to be more conspicuously urban in structure and influence. At the least, this example illustrates the kind of pervasive influence that Lefebvre envisioned for the urban over the rural (Lefebvre 2003). See the paper by Pickett et al. (2024) on the Relational Shift in Urban Ecology: From Place and Structure to Multiple Modes of Coproduction for Positive Urban Futures, in this Special Feature, for more discussion of urbanity beyond the city

## Challenges and opportunities of the transformative shift in urban ecology

### Challenges

Co-production incorporating urban ecological knowledge comes with challenges (Pathway #1). Safeguarding institutional spaces for this mode of knowledge enterprise requires time, effort, and governance capacity that are typically only standard practice in some cities, especially in medium-sized and small cities (Patel 2022). Scoggins et al. (2022) pointed to the power and trust differentials among planners, experts, and citizens in restoration projects, and noted that institutional settings often restrict a shift to more equitable participation and engagement approaches. Another challenge is that of language and vernacular (i.e., terms and jargon used by participants are not always aligned nor common) and speed difference among scientists, planners, residents, and others in terms of synchronicity between policy cycles and scientific discovery (also mentioned in Frantzeskaki et al 2019). This requires time to adjust to each other’s worlds, to truly connect, and to ensure that everyone is talking about the same topics and issues. To overcome these challenges, urban ecology must continue building from knowledge and experience of other disciplines, mainly those with longstanding expertise with collaborative research (such as Participatory Action Research) and innovating transdisciplinary inquiry, such as sustainability science (Pereira et al. 2019). Such cross-over with other disciplines will continue strengthening urban ecology research as it adopts community-based approaches and collaborative research (Gordon et al 2019; Boone et al 2020). This "ecology for and with cities" push is mainly directed at urban planning and urban landscape architecture, with recent research efforts to integrate other disciplines, such as design thinking to inform transdisciplinary inquiry of urban ecology with cities (Marshall et al. 2020). Knowledge and thinking from disciplines that are focused on social and environmental justice and equity also needs to be firmly embedded in the knowledge → action → knowledge spiral (Schell et al 2020; Pickett and Grove 2020; Roberts et al 2022).

Another key challenge pertains to integrating SETS thinking and approaches into urban planning (Pathway #2). In most planning processes, social, ecological, and technological components have traditionally been pursued separately (Mehvar et al. 2021) and their integration has not received enough attention. What role should urban ecologists play in promoting these integrative and systemic SETS approaches? The arguments have been made that urban ecologists need to become more informed about and involved in practices such as design (Pickett et al. 2022; Childers et al. 2015) and planning (Grove et al. 2017, 2018), that design and planning need to consider multiple ecosystem services (Andersson et al. 2015; Meerow and Newell 2017), and that planning should include ecological performance evaluation (Cortinovis and Geneletti 2020). Urban ecologists have now started to engage with engineering (Markolf et al 2018; Chester et al 2023), which is fundamentally responsible for most urban built infrastructure. Many engineered and built solutions are becoming less reliable and more vulnerable to systemic failures and collapse precisely because they are based on concepts of rigidity, inflexibility, and experience from the past. These built solutions were designed and built based on past conditions and are often unprepared for the uncertainties of current and future climate change pressures. Their prevalence is an effect of institutional lock-in that posits them as the preferred options for urban infrastructure development, with seemingly little consideration of urban sustainability or urban resilience goals (Buzási and Csizovszky 2023).

### Opportunities

In order to advance and accelerate this transformative shift of urban ecology, we need to educate future urban ecologists to be inter- and transdisciplinary thinkers and professionals using new pedagogical and mentoring practices and paradigms that go far beyond the traditional paradigms of linear connections from knowledge to policy/planning and society. This will require changes in scientific practices, including long-held beliefs about scientific objectivity, in relations between scientists and communities and planners, and in assumptions about science-society boundaries, epistemological inclusivity, and exclusivity. This is also relevant to research and practice of nature-based solutions in cities, where the need for more inclusive and open science approaches have been advocated as needed to deal with their design and implementation for more just urban futures (Raymond et al 2023; Tozer et al 2020; Wickenberg 2023). Specifically, the urban sandbox in Fig. 1 is the venue for transdisciplinary design of nature-based solutions that is centered on collaborative research designs and open science principles. This will open the process to new knowledges, experiences, and solutions to co-produce sustainable and resilient urban pathways based on nature-based solutions, expanding toward nature-based urbanism futures. This will not only enhance inclusivity in the planning and governance of nature-based solutions (Kabisch et al 2022) but it will also strengthen the place-suitability of selected nature-based solutions (Croeser et al 2021). In a real sense, this is a call to decolonialize the traditional Western approaches to and ways of thinking about science, including urban ecology.

To be part of this transformation shift, urban ecologists will need to transform themselves from scientist-researchers into researcher-practitioners and play a stronger role in initiating dialog with designers, planners, and decision-makers. Co-production contributes to the motivation of citizens but also practitioners in the planning, design, and stewardship of urban ecosystems and thus helps to strengthen the initiatives and their impact on society. This will require a more reflexive practitioner stance in the knowledge enterprise that can center on internalizing different ways of generating knowledge. This will require moving beyond recognizing the positionality of urban ecologists as well as their contribution to co-production that incorporates urban ecological knowledge with other knowledges. The inverse is also needed: An education and training of urban planners and other co-production practitioners on how to work with scientists, raising awareness of what is needed and what can be gained from it.

An additional opportunity to accelerate this transformation shift comes from the increasing advances in information and communication technologies that intersect with urban ecology. These advances, including the widespread use of sensors and wearable devices, have facilitated the development of urban tools and platforms that streamline interactions across social, ecological, and technological domains. Among other things, such tools and platforms can enhance the efficacy and efficiency of urban operations, increase adaptive capacity to unpredictable climate-induced stressors, enable real-time response to emerging needs, facilitate enhanced visioning and scenario planning, and foster bottom-up engagement of stakeholders in planning and environmental stewardship initiatives (Ward et al. 2019; Li and Nassauer 2021; Wellmann et al., 2023). These new technologies can also benefit urban ecologists by connecting them with various actors—members of the urban sandbox—and incorporating the knowledges of previously marginalized actors. Achieving these benefits, however, hinges on ensuring that smart systems and technological solutions are inclusive and do not create new forms of inequalities in society.

## Conclusion

We began with a brief review of the expansion of urban ecology from an ecology *in* cities to an ecology *of* cities, and to what is now an ecology *for* or *with* cities. In this transdisciplinary phase, urban ecology is continuing to transform as a boundless field. We see urban ecology growing and maturing as the transformative shift we present here opens scientific inquiry to other experts, to planners, and to citizens and communities—to an all-inclusive urban sandbox of willing actors. We see this transformative shift through the three pathways, and we present a framework for how these pathways intertwine. The first pathway builds on existing good practices in urban ecology that are centered collaboration and co-production and growing them into a knowledge → action → knowledge spiral that is constantly iterating in response to new problems and challenges, and is constantly producing sustainable and resilient solutions. To demonstrate the efficacy of this spiral, we presented two case studies on the co-production of transformative solutions in the Stiemervalley, Belgium, and on inclusive, just co-production of solutions for the Bronx, New York. The second pathway positions interdisciplinary SETS approaches as central to the knowledge → action → knowledge spiral. The second pathway will strengthen the resilience of urban infrastructures and decision-making processes as it spirals into the realm of deep futures in a temporal extension that acknowledges both the uncertainties and the opportunities that the future holds. We demonstrate the value of this focus on SETS approaches and on the deep future with a case study of the development of future urban climate resilient scenarios for New York City. The third pathway in our transformative shift framework expands urban ecology with a renewed focus on rural areas and places that are not yet urban, acknowledging that roughly half of the places that will be cities in 2050 are not yet urban. Our fourth case study, from the Yanqi Valley in China, demonstrates the opportunities for transformative urban change beyond places that are currently cities, with their embedded systemic inertias that often fight real change. Finally, we recognize the importance of weaving equity and justice throughout the entire transformative shift framework as we continually work to undo the legacies of discrimination in our cities and of colonialist thinking in the science of urban ecology.
